# “Aria di Ricerca in Valle del Serchio”: a cross-sectional citizen science study to evaluate CKD prevalence and associations with environmental risk factors in the Serchio Valley (Lucca, Tuscany, Italy)

**DOI:** 10.3389/fpubh.2025.1536070

**Published:** 2025-05-21

**Authors:** Chiara Doccioli, Francesco Sera, Giorgia Stoppa, Bruna De Marchi, Dolores Catelan, Antonella Ficorilli, Giulia Malavasi, Annibale Biggeri

**Affiliations:** ^1^Clinical Epidemiology Unit, Institute for Cancer Research, Prevention and Clinical Network–ISPRO, Florence, Italy; ^2^Department of Statistics, Computer Science and Applications G. Parenti, University of Florence, Florence, Italy; ^3^Unit of Biostatistics, Epidemiology and Public Health, Department of Cardiac, Thoracic, Vascular Sciences and Public Health, University of Padova, Padova, Italy; ^4^Epidemiologia e Prevenzione "Giulio A. Maccacaro" Social Enterprise, Milan, Italy; ^5^SVT, University of Bergen, Bergen, Norway; ^6^La Sapienza University, Rome, Italy

**Keywords:** chronic kidney disease, estimated glomerular filtration rate (eGFR), citizen science, environmental contamination, Italy

## Abstract

**Background:**

Chronic kidney disease (CKD) represents a growing global public health issue, with an estimated prevalence of around 11% in the most developed countries. This study, conducted as part of the European project CitieS-Health, aimed to estimate the prevalence of CKD in the Serchio Valley, Tuscany, Italy, an area subject to environmental contamination from various sources, including a potentially polluting copper foundry.

**Methods:**

This cross-sectional study was conducted from 2019 to 2022 among a representative sample of 400 adults from eight municipalities, integrating a citizen science (CS) approach to enhance public engagement. The study aimed to estimate the prevalence of CKD in the area, as assessed by the decrease of estimated Glomerular Filtration Rate (eGFR). Data on lifestyle, clinical parameters, and environmental exposures were collected, employing a standardized protocol developed by the Disadvantaged Populations eGFR Epidemiology Study (DEGREE). The eGFR was calculated using three equations: CKD-EPI 2009, Modification of Diet in Renal Disease (MDRD), and CKD-EPI 2021. Associations between risk factors and CKD were examined through multivariate analyses.

**Results:**

Results revealed a CKD prevalence of 12.7% using the CKD-EPI 2009 formula, 15.8% with the MDRD equation, and 10.5% according to the CKD-2021 definition, with age, hypertension, and diabetes being significant risk factors. These estimates are significantly higher than the national average reported in Italian studies (6–9%). Moreover, residential proximity to (< 2 km) and employment in the copper foundry resulted associated with eGFR reduction (OR = 1.36; 90%CI = 0.80, 2.29 and OR = 2.14; 90%CI = 0.89, 5.13, respectively; estimated with ordinal logistic regression, CKD defined per 2021 criteria).

**Conclusion:**

In conclusion, the study revealed an increased prevalence of CKD in an area affected by heavy metal pollution, particularly cadmium. These findings underscore the impact of environmental exposures on kidney health, emphasizing the need for targeted interventions and public health measures to mitigate CKD prevalence in pollution-exposed communities.

## 1 Introduction

Chronic kidney disease (CKD) is a condition characterized by the presence of kidney damage lasting for more than 3 months (1). CKD is a global public health problem with a growing prevalence worldwide (2). According to the 2019 Global Burden of Disease study, CKD was ranked 29th on the list of causes of global disability-adjusted life-years (DALYs) in 1990, but rose to 18th place in 2019 (3, 4). The global burden of CKD is rapidly increasing and is projected to become the 5th most common cause of years of life lost worldwide by 2040 (5). Several studies on CKD prevalence have been performed globally. Although different study designs and inconsistent definitions of CKD have been used, the prevalence of CKD in more economically developed countries has been consistently estimated at around 11% (6). In Europe, Brück et al. (7) collected CKD prevalence data from 19 general population studies conducted in 13 European countries. The authors highlighted a strong heterogeneity among the different European populations, with prevalence ranging between 3.3% in Norway and 17.3% in North-East Germany. The Italian INCIPE study estimated a prevalence of CKD stages 1–5 of 9.6% (8). A subsequent Italian study (9) estimated a prevalence of CKD for all stages of 6.3%. International comparisons have provided the first steps in understanding CKD, but such comparisons rely on the availability of standardized tools to estimate disease prevalence. This is a real concern in CKD, since the disease is asymptomatic until the late stages, and the biases inherent in the methods used to estimate the glomerular filtration rate (GFR) in population studies are highly variable across populations. The Disadvantaged Populations estimated Glomerular Filtration Rate (eGFR) Epidemiology Study (DEGREE) (10) is an international collaboration study whose goal is to estimate distributions of renal function globally, especially in disadvantaged communities, using a standardized study protocol based on the estimation of GFR from serum creatinine value. This epidemiological effort is aimed to quantify the extent of CKD adjusting for known risk factors – hypertension, diabetes, proteinuria – which could be linked in selected populations to previously unknown risk factors, including heavy metals. In fact, there is increasing recognition of forms of CKD that are not associated with traditional risk factors, but rather with environmental exposures such as heat stress, dehydration, pesticides, and heavy metals (11, 12).

Citizen Science (CS) is a rather new entry in the health and epidemiological fields, participatory research is gaining increasing interest and credit despite the recognition of the challenges it entails. Indeed “it has been found to increase the relevance of research questions, result in better knowledge production, and impact research policies” (13).

However, though CS has become more common in environmental monitoring, its use in environmental epidemiology remains limited. This approach is particularly valuable in contexts of intense environmental pressure, where community cohesion has been weakened. In such settings, a lack of trust can lead to low participation rates, particularly in studies involving biomonitoring (14).

The present study aimed to estimate CKD prevalence in the Serchio Valley (province of Lucca, Tuscany, Italy), an area that suffers from environmental contamination from various sources, following the DEGREE standardized protocol and the Citizen Science approach developed in the CitieS-Health project.

## 2 Methods

The present study draws on two international initiatives: the DEGREE study and the CitieS-Health project. The DEGREE study was designed to facilitate international comparisons of eGFR distribution. The collaboration has produced a standardized protocol for estimating the worldwide population distribution of eGFR. The project involved quantifying renal function in a representative adult population-based sample with standardization of serum creatinine measurements, along with storage of samples for future measurements and ascertainment of body composition estimates. The DEGREE methodologies are described in detail elsewhere (10).

CitieS-Health (15, 16) is a CS EU funded project in environmental epidemiology conducted in five European countries: Italy, Lithuania, the Netherlands, Slovenia and Spain. The project adopted a Post-Normal Science (PNS) framework, which is considered appropriate and effective in situations when “facts are uncertain, values in dispute, decision-stakes high, and decisions urgent” (17). The focus shifts from finding the true, undisputable results to assuring the quality of the process of investigation. This requires the construction of an “extended peer community” (EPC) including multiple actors with diverse types of knowledge and expertise, disciplinary and not (17). The ambition is that all those involved will be willing and capable to address issue of common interest and/or concern listening to each other with mutual curiosity and respect.

In particular, the CitieS-Health project adopted a broad understanding of CS, closer to Irwin’s (1995) (18) than to Bonney’s (19), the two authors that independently coined the expression in the mid-1990s. Indeed, the “lay participants” in the project are considered on an equal standing with the professional researchers and have a voice in all stages of the investigation: the selection of the research questions, the design of the research protocols, the collection of data, their analysis and interpretation, the formulation of conclusions, the communication of results to multiple audiences through co-authored scientific articles, policy briefs, documents and materials for wide dissemination, etc.

The present study – the Italiana case study of the CitieS-Health project - study was conducted in the Serchio Valley, an area of natural, cultural and historic significance but also exposed to different sources of environmental pollution, including from industrial activities. The research team comprised members with a variety of backgrounds and specializations ranging from the medical-health field to the social sciences and the humanities, to ethics. They all agreed on the purpose of maximizing the involvement of residents in all the phases of the project.

### 2.1 Study design and participants

Our study was conducted from January 2019 to September 2022 in the Serchio Valley (20), namely in the municipalities of Barga, Borgo a Mozzano, Coreglia Antelminelli, Fabbriche di Vergemoli, Fosciandora, Gallicano, Molazzana and PieveFosciana. The Serchio Valley area suffers from environmental pollution from a diverse range of sources, including a potentially polluting copper foundry located in the municipality of Barga, which has long been a critical health concern for the local residents. Thus, the idea of a study like ours was welcomed, in particular by many members of 18 local associations who became very active in diffusing information and promoting participation. In particular, they helped in the organization of a sociological survey dedicated to detecting knowledge, opinions and perceptions about health and environmental conditions in the Valley. Of the 1,052 questionnaires distributed, as many as 915 valid ones were returned. The results were presented in a public meeting, also attended by local mayors and administrators, generating a profitable debate and increasing the motivation to participate in the more strictly epidemiological part of the study. The public health concern, in particular, was the industrial settlement that seems to be potentially linked to heavy metal pollution – including cadmium - leading to soil and water contamination (21). In addition, historical research was conducted to identify possible sources of environmental pressure over the past decades. Official documents, grey literature and interviews with key witnesses provided a broad picture of the deep influence of the copper foundry (active since 1916) on all aspects of the life of the local communities: economic choices, employment, education, social and cultural habits and conventions, and so on (22). Again, the purpose was to maximize the engagement of the local population in order to better interpret environmental and health data, shedding light on both past and present events and contingencies. In the same vein, air quality measurements were conducted by means of self-built sensors installed by the citizens.

The study sample consisted of adults aged ≥18 years, resident in one of the eight municipalities of the Serchio Valley at the date of 1^st^ January 2020, selected through random sampling stratified by age and sex from municipal resident registries. Sampled subjects were invited to participate with a letter. Since the CKD prevalence varies among the different age classes, we considered different sampling fractions in order to have about 10 cases for each age group (age groups 18–39; 40–49; 50–59; 60–69; 70+).

In particular, to obtain a non-negligible number of cases in the younger age groups, we proportionally decreased the sample size (see Supplementary Appendix A.2, Table A.2.1). Subjects in conditions limiting their ability to express free informed consent were excluded from the study.

The study protocol and the related documents were approved by the Research Ethics Committee of the University of Florence in March, and by the Medical Ethical Committee of Tuscany Region, section North-West Area in April 2021 (23). The rationale for this two-step approval process can be found in a recently published article by Ficorilli et al. (24) Informed written consent was obtained from all the participants enrolled.

### 2.2 Data collection and laboratory measurements

In the mind of co-creation and co-design, the research team collaborated with the citizens and the local health professionals to set up a dedicated outpatient clinic where participants’ biospecimens were collected and stored. Some local citizens, health professionals, and biologists were recruited and appropriately trained to collect data and biological samples (25).

In order to pursue the study’s aim, data were collected through two consecutive steps: the first step implied the administration of an *ad hoc* questionnaire on lifestyle and food consumption habits and occupational history, via remotely ICT supported telephone interviews. In the second step participants were invited for a visit to obtain basic clinical data and to collect and store blood and urine samples.

The clinical evaluation included blood pressure measurement taken after 5 min of resting position (seated) by a manual sphygmomanometer; stand height (cm) by a wall stadiometer; body weight (kg) and body composition (i.e., body fat percentage) by a bioimpedance analyzer balance (TANITA MC-780MA).

### 2.3 Analysis of biological samples

The participants’ biospecimens and data were stored and managed by the professional research team, based on the conditions specified in the epidemiological protocol, for the whole duration of the CitieS-Health project. According to the study protocol, after the end of the project the biological samples were transferred to a central laboratory in the University of Udine for centralized analyses with standard methods.

Urine dipstick analyses yielded semi-quantitative blood, proteins, leukocytes, glucose, pH and urine density values. Serum creatinine was measured in 5 mL of serum sample. Serum samples were stored for future determinations as in the DEGREE Study protocol. According to the co-created Citizen Science approach, specific rules were defined to guarantee donors’ rights to participate in the research (26).

We calculated the estimated Glomerular Filtration Rate (eGFR) using the Modification of Diet in Renal Disease (MDRD) equation (27) as well as the 2009 and 2021 Chronic Kidney Disease Epidemiology Collaboration (CKD-EPI) formulas (hereafter referred to as the CKD-EPI 2009 equation and the CKD-EPI 2021 equation, respectively) (28, 29).

We defined five categories of kidney function based on eGFR results: G1, normal or high (≥90 mL/min/1.73 m^2^); G2, mildly decreased (60–89 mL/min/1.73 m^2^); G3a mildly to moderately decreased (45–59 mL/min/1.73 m^2^); G3b moderately to severely decreased (30–44 mL/min/1.73 m^2^); G4, severely decreased (15–29 mL/min/1.73 m^2^); and G5, kidney failure (<15 mL/min/1.73 m^2^). CKD was defined and classified based on KDIGO guidelines as the proportion of individuals with moderate or established kidney function (eGFR < 60 mL/min/ 1.73m^2^) in the overall sample.

### 2.4 Questionnaires

During the previously mentioned ICT-supported telephone interview, questionnaires were used to gather information on socio-demographic factors (age, sex) and socioeconomic status (education, employment status, household income). Environmental conditions associated with CKD (previous or current work in the copper foundry plant) were also addressed. Information on past medical history was focused on self-reported cardiovascular and chronic diseases (arterial hypertension, diabetes, myocardial infarction or other heart diseases, chronic respiratory diseases, cirrhosis), and behavioral risk factors (dietary habits, alcohol consumption, smoking, and physical activity). We also included questions on CKD and its associated causes, including history of congenital kidney malformation, diabetic nephropathy, polycystic kidney disease, urolithiasis, and glomerulonephritis.

### 2.5 Statistical analysis

For the sample size estimation, reference was made to previous CKD prevalence studies conducted in Italy, particularly the INCIPE (8) and the CARHES study (9). These studies documented a prevalence range between 6 and 9%.

To estimate the sample size, we used the following formula (30): n = z^2^ × p × q/b^2^, where.

- z = standard normal deviation value at the desired confidence level (CI). For CI = 90%, z = 1.645.
- p = expected prevalence (7.5%).
- q = 1-p.
- b = desired accuracy. In our case, 1.5%.

The sample size calculation gave a sample size equal to 834, subsequently increased to 1,000 to meet the DEGREE protocol’s requirements. We aimed to recruit 1,000 subjects and selected 1,000 quadruplets through age and sex stratified sampling, from the demographic records of the local municipalities. Due to the COVID-19 pandemic, mailing invitations was impracticable, leading to the adoption of a door-to-door delivery method.

Ultimately, we succeeded in recruiting 500 individuals, with 41 refusing to participate. The present study analyses the first 400 samples, collected as part of the European project, with the recruitment phase starting in March 2021 and concluding temporarily in April 2022, earlier than the originally planned end date in June 2022. According to the standards of the American Associations for Public Opinion Research (31), our minimum response rate (RR1) was 50%. This rate includes the number of interviews (complete and partial) plus the number of non-interviews (refusal and break-off plus non-contacts plus others) plus all cases of unknown eligibility (including mailing failures) in its denominator. However, in survey research, the response rate—also known as the completion rate or return rate—is calculated by dividing the number of respondents by the sample size. In our case, the response rate is 92%.

In the descriptive analysis, categorical variables were described by their absolute and relative frequencies, while continuous variables were described using mean ± standard deviation (SD). Group comparisons were performed using the chi-square test for categorical variables and the t-test for continuous variables.

The prevalence of CKD and the relative 90% confidence intervals were estimated through stratified estimators that considered post-stratified weights calculated considering the different sampling fractions, and response rate by age and sex (See Supplementary Appendix A.3, Table A.3.1). Using the post-stratified estimators, the prevalence and relative confidence intervals were also estimated for each of the stages 1–5 of the KDIGO classification (32).

We examined differences in CKD prevalence according to age groups and between men and women. We used a Student’s t-test to examine the difference between the two sexes and we performed a linear regression to compare the different age classes.

We used linear regression models (with regression coefficient and 90% confidence intervals) to estimate associations between potential risk factors and continuous eGFR, and ordinal logistic regression (with Odd Ratio, OR, and 90% confidence intervals) to estimate associations between risk factors and reduced eGFR. Adjusted models included age, sex, diabetes, hypertension, residential proximity to the copper foundry and working in the copper foundry. To enhance the stability of the estimates, given the small number of events observed within certain age groups, we dichotomized age at the median in multivariate analyses. The models were fitted with post-stratified weights calculated considering the sampling fraction and response probability, stratified by age and sex, to produce estimates representative of the population covered by the study. As referent population we consider the population resident in the municipality of Barga according to the 2020 Census.

A kernel density estimate was used as an exploratory analysis to describe the sampling density of the invited, the respondent, and the ratio of respondent/invited for the survey in space *λ*(s) (33).

All analyses were performed using STATA/SE version 17.0 (Stata Corp., College Station, TX) and RStudio (version 2023.09.1 + 494) statistical software.

## 3 Results

### 3.1 Demographic and clinical characteristics of participants

From March 2021 to April 2022, 400 individuals aged between 18 and 86 years accepted to participate in the present study. Table 1 presents the sample’s socio-demographic characteristics. Over half of the participants were > 50 years old and 55.5% were females. The smoking rate was 16.5%, slightly higher in men (16.7%) than women (16.3%). The prevalence of reported arterial hypertension was 20.0%. Seventy-five percent of the population completed ≥10 years of formal education. Using the BMI values, a prevalence of 38% for overweight and 15.7% for obesity were detected. A total of 16 (4.1%) participants reported a previous diagnosis of diabetes, and 300 (75.6%) referred to be alcohol drinkers. Moreover, 22.7% of the study population lived close to the copper foundry (<2 km distance), and 25 subjects (6.2%) reported having worked in the foundry (Table 1). To consider the difference in response rate among the various age classes and between sexes (see Supplementary Appendix A.3, Table A.3.1), and the oversampling of females and younger ages, subsequent analyses were weighted to be representative of the population.

**Table 1:** Socio-demographic and clinical characteristics of study participants in the study group and by sex.

Characteristics	All (400)	Men (178)	Women (222)
	*N* (%) or mean ± SD	*N* (%) or mean ± SD	*N* (%) or mean ± SD
Age, years	49.5 ± 15.5	52.1 ± 15.3	48 ± 15.4
Age class			
18–39	100 (25.0%)	37 (20.8%)	63 (28.4%)
40–49	72 (18.0%)	27 (15.2%)	45 (20.3%)
50–59	123 (30.8%)	62 (34.8%)	61 (27.5%)
60–69	70 (17.5%)	30 (16.8%)	40 (18.0%)
70+	35 (8.7%)	22 (12.4%)	13 (5.8%)
Alcohol drinkers	300 (75.6%)	149 (84.7%)	151 (68.3%)
Education level			
Primary school (5 years of schooling)	14 (3.5%)	6 (3.4%)	8 (3.6%)
Middle school (8 years of schooling)	84 (21.2%)	50 (28.4%)	34 (15.5%)
High school (13 years of schooling)	175 (44.2%)	79 (44.9%)	96 (43.6%)
University (≥16 years of schooling)	123 (31.1%)	41 (23.3%)	82 (37.3%)
BMI, kg/m^2^	25.6 ± 4.7	27.0 ± 4.0	24.6 ± 4.9
BMI class			
Underweight (<18.5)	11 (2.7%)	1 (0.6%)	10 (4.5%)
Normal weight (18.5–24.9)	173 (43.3%)	53 (29.8%)	120 (54.0%)
Overweight (25–29.9)	153 (38.3%)	88 (49.4%)	65 (29.3%)
Obesity (>30)	63 (15.7%)	36 (20.2%)	27 (12.2%)
Current smokers (active smoking)	65 (16.5%)	29 (16.7%)	36 (16.3%)
Diabetes	16 (4.1%)	9 (5.1%)	7 (3.2%)
Arterial hypertension	79 (20.0%)	45 (25.6%)	34 (15.6%)
Residential proximity to the copper foundry (< 2 km)	91 (22.7%)	40 (22.5%)	51 (23.0%)
Working in the copper foundry	25 (6.2%)	22 (12.3%)	3 (1.3%)

### 3.2 Prevalence of CKD

The DEGREE protocol specifies a cut-off of eGFR < 60 mL/min per 1.73m^2^ for CKD, namely for defining reduced kidney function. In the overall population sample, accordingly to the CKD-EPI 2009 formula, the weighted prevalence was 12.7% (90%CI = 8.9, 18.4 based on 24 subjects) for an eGFR < 60 mL/min/1.73m^2^ compatible with the definition of CKD, whereas 211 subjects (weighted prevalence 51.8%; 90%CI = 47.0, 56.5) had an eGFR ≥ 60 but < 90 mL/min/1.7m^2^. Instead, according to the MDRD formula, 35 cases (15.8%; 90%CI = 11.7, 21.6) had an eGFR < 60 mL/min/1.73m^2^ compatible with the definition of CKD, whereas 282 (65.4%; 90%IC = 60.3, 70.1) had an eGFR ≥60 but < 90 mL/min/1.73m^2^. Finally, according to the CKD-EPI 2021 formula, 18 cases (10.5%; 90%CI = 7.2, 15.1) had an eGFR < 60 mL/min/1.73m^2^, while 170 (44.4%; 90%CI = 42.2, 49.2) had an eGFR between 60 and 90 mL/min/1.73m^2^ (Table 2). This prevalence is significantly higher than the average prevalence estimates reported in the Italian studies (8, 9).

**Table 2:** Prevalence of eGFR categories using KDIGO Guidelines with CKD-EPI 2009, 2021 and MDRD formula.

eGFR (ml/min/1.73m^2^)	Estimated prevalence					
	*N*	CKD-EPI equation (90%CI)	*N*	MDRD equation (90%CI)	*N*	CKD-EPI 2021 equation (90%CI)
≥90	164	35.5% (32.8, 38.2)	82	18.9% (16.4, 21.7)	212	45.2% (42.2, 48.2)
60–89	211	51.8% (47.0, 56.5)	282	65.4% (60.3, 70.1)	170	44.4% (42.2, 49.2)
30–59	23	12.4% (8.9, 16.9)	34	15.5% (11.7, 20.1)	18	10.5% (7.2, 15.1)
15–29	1	0.3% (0.05, 1.5)	1	0.3% (0.005, 1.5)	0	–

Values adjusted for sampling fraction and response probability.

The mean eGFR was 81.9 ± 15.4 mL/min/1.73m^2^ when calculated with the CKD-EPI 2009 equation, 85.6 ± 15.9 with the CKD-EPI 2021 and 75.5 ± 15.2 mL/min/1.73m^2^ with the MDRD formula (Table 3). In particular, we observed a lower eGFR with increasing age and in the female sex regardless of the equation used to calculate it.

**Table 3:** Distribution of baseline eGFR with all formulas.

Variables	*N*	eGFR estimates						
		CKD-EPI equation		MDRD equation		CKD-EPI 2021 equation		Values from the literature
		Mean ± SD	*p*-value	Mean ± SD	*p*-value	Mean ± SD	*p*-value	Mean
Overall	400	81.9 ± 15.4		75.5 ± 15.2		85.6 ± 15.9		
Sex								
Male	178	83.7 ± 15.6	0.0179^a^	78.3 ± 16.0	0.0004^a^	87.7 ± 24.3	0.0111^a^	
Female	222	80.1 ± 14.7		72.8 ± 14.0		83.6 ± 20.6		
Age class								
18–39	100	103.3 ± 13.0	<0.0001^b^	90.8 ± 14.6	<0.0001^b^	106.04 ± 26.4	<0.0001^b^	110
40–49	72	88.1 ± 14.7		78.6 ± 14.0		91.7 ± 35.5		99
50–59	123	83.3 ± 11.1		76.6 ± 11.9		87.3 ± 20.7		93
60–69	70	76.9 ± 12.5		73.4 ± 12.6		81.5 ± 30.7		85
70+	35	60.2 ± 12.7		59.9 ± 12.3		64.3 ± 45.7		75

a *T*-test.

b Regression.

Values adjusted for sampling fraction and response probability.

The most important risk factors for CKD (diabetes and hypertension) were evaluated in the sample. In addition, we considered mean glomerular filtration rates for those who lived close to the copper foundry and those who had worked in such industries (Table 4). Regarding work history and residence, we observed a GFR reduction of 4.5 and 2.5% respectively: a less evident result than for the well-known risk factors but still worthy of attention and further exploration.

**Table 4:** Absolute and percentage GFR reduction (CKD-EPI 2021 equation) for each kidney disease risk factor (diabetes and arterial hypertension) and for subject characteristics (work history in the copper foundry and residence in the vicinity of the copper foundry).

Variables	Estimated glomerular filtration rate	
	Absolute difference (Yes-No)	CI 90%
Diabetes (Yes vs No)	15 (7.5%)	5.20 (2.5, 10%)
Arterial hypertension (Yes vs No)	20 (10%)	10.22 (5, 11%)
Work history (Yes vs No)	9 (4.5%)	4.14 (2, 7%)
Distance <2 km (Yes vs No)	5 (2.5%)	3.9 (1.5, 4.5%)

Sample of 400 residents in the Serchio Valley 2021–2022. 90% confidence interval.

In multivariate linear regression analysis, diabetes and hypertension were negatively associated with eGFR (Table 5). In the fully adjusted linear regression model (2021 CKD-EPI equation), age above 52 years emerged as a key risk factor for reduced eGFR. In fact, when considering age above 52 as the reference category, the regression coefficient for age equal to or below 52 was 16.5 (90% CI = 13.70, 19.34). Residential proximity to the copper foundry and working in the copper foundry were both associated with lower eGFR (regression coefficient = −3.72 90%CI = −7.54, 0.09; and regression coefficient = −4.48 90%CI = −9.76, 0.81, respectively). Female sex was also associated with lower eGFR (regression coefficient = −4.93; 90%CI = −7.56, −2.31).

**Table 5:** Multivariate linear regression analysis of risk factors for reduced eGFR (continuous).

Variables	Model – CKD-EPI equation	Model – MDRD equation	Model – CKD-EPI 2021 equation
	Coeff. (90% CI)	Coeff. (90% CI)	Coeff. (90% CI)
Age class			
> 52 years	ref	ref	ref
18–52 years	17.67 (14.92, 20.43)	10.61 (7.97, 13.25)	16.5 (13.70, 19.34)
Female sex	−4.62 (−7.19, −2.06)	−5.92 (−8.45, −3.40)	−4.93 (−7.56, −2.31)
Diabetes	−4.36 (−11.16, 2.43)	−4.43 (−10.92, 2.05)	−4.46 (−11.55, 2.62)
Arterial hypertension	−9.44 (−13.56, −5.34)	−7.51 (−11.26, −3.77)	−9.71 (−13.98, −5.45)
Residential proximity to the copper foundry (< 2 km)	−3.85 (−7.58, −0.12)	−3.93 (−7.31, −0.56)	−3.72 (−7.54, 0.09)
Working in the copper foundry	−4.67 (−7.19, −2.06)	−2.54 (−7.45, 2.37)	−4.48 (−9.76, 0.81)

Model: age, sex, diabetes, hypertension, BMI, residential proximity to the copper foundry, working in the copper foundry, sampling fraction and response probability.

Moreover, consistent results were obtained when applying the alternative eGFR estimation equations.

ORs for the association between risk factors and CKD at multivariable ordinal logistic regression analysis are reported in Table 6. In the fully adjusted logistic regression model, using the CKD-EPI 2021 equation, the odds of reduced eGFR increased with diabetes (OR = 1.11; 90%CI = 0.42, 2.93) and hypertension (OR = 2.94; 90%CI = 1.69, 5.12). Age was a key risk factor for lower eGFR, especially for the class 52 + (OR for the class 18–52 = 0.16; 90%CI = 0.11, 0.23).

**Table 6:** Multivariate ordinal logistic regression analysis of risk factors for CKD.

Variables	Model – CKD-EPI equation	Model – MDRD equation	Model – CKD-EPI 2021 equation
	OR (90% CI)	OR (90% CI)	OR (90% CI)
Age class			
> 52	1	1	1
18–52	0.17 (0.11, 0.24)	0.24 (0.15, 0.38)	0.16 (0.11, 0.23)
Female sex	1.55 (1.04, 2.32)	1.87 (1.21, 2.89)	1.87 (1.24, 2.83)
Diabetes	2.38 (0.63, 9.03)	2.04 (0.53, 7.82)	1.11 (0.42, 2.93)
Arterial hypertension	4.34 (2.36, 7.97)	2.96 (1.54, 5.68)	2.94 (1.69, 5.12)
Residential proximity to the copper foundry (< 2 km)	1.39 (0.85, 2.28)	3.06 (1.79, 5.22)	1.36 (0.80, 2.29)
Working in the copper foundry	1.35 (1.07, 5.12)	1.38 (0.62, 3.09)	2.14 (0.89, 5.13)

Model: age, sex, diabetes, hypertension, BMI, residential proximity to the copper foundry, working in the copper foundry, sampling fraction and response probability.

In the fully adjusted model, residential proximity to the copper foundry (OR = 1.36; 90%CI = 0.80, 2.29) and working in the copper foundry (OR = 2.14; 90%CI = 0.89, 5.13) were factors associated with impaired kidney function (Table 6, CKD-EPI 2021 equation).

When employing alternative eGFR estimation equations, the findings remained consistent.

## 4 Discussion

The prevalence of CKD of stage 3 (eGFR < 60 mL/min per 1.73m^²^) in our sample was 12.7% (24 cases) according to the CKD-EPI 2009 formula, 15.8% (35 cases) according to the MDRD, and 10.5% (18 cases) based on the CKD-2021 definition. We used both the CKD-EPI and the MDRD formulae as suggested by the DEGREE protocol. The first CKD-EPI formula was introduced in 2009 to address some of the limitations of the MDRD equation: in particular, one of the drawbacks of the MDRD equation is that it does not accurately classify patients with early CKD, particularly those with values >60 mL/min/1.73m^2^ (34). In 2021, the joint task force led by the American Society of Nephrology and the National Kidney Foundation came up with a new CKD-EPI equation without the race factor and recommended adopting this equation (based on CKD-2009). In studies of populations living in areas exposed to heavy metals, average eGFR values varied between 88 and 120 mL/min/1.73m^2^ (35–38).

Overall, the prevalence of CKD observed in our study was higher than the 6.3% prevalence reported from the Italian CARHES study (9) and the 9.6% reported in the INCIPE study (8).

Several factors have been shown to impact the overall glomerular filtration rate: ageing, hypertension, diabetes, obesity and several dietary patterns have a decreasing direct and indirect effect on GFR (39, 40).

In our study, the most important risk factors for CKD (diabetes and hypertension) (41) were evaluated in the sample. Moreover, we considered living close to the copper foundry and having worked in such industries (12). Therefore, we documented the existence of a hazard for those who worked in the copper foundry and an increased risk for those who live close to the foundry.

Regarding the plausibility of our results (Table 4), it is interesting to note that the kidney has an important functional reserve: in optimal conditions (young age and absence of concomitant diseases) the kidneys filter about 150–200 liters of plasma daily, and kidney disease is clinically manifested when filtration is reduced to 20 liters per day. Our results showed that diabetes and hypertension lead to a reduction in the optimal functional reserve of around 10%, which can become clinically relevant when the renal functional reserve is reduced with age. The results of our study are particularly relevant for the local community. For a broader interpretation in the context of the DEGREE initiative and international comparisons, readers are referred to the recent global analysis published within the DEGREE programme (11). Future research is planned to explore the effect of dietary factors and the association between eGFR and heavy metals’ serum concentration.

Higher hospitalization rates for CKD had emerged as a public health concern in the Serchio Valley in 2018 following an investigation by the Regional Health Agency of Tuscany. Studies conducted to date are consistent in highlighting an unfavorable epidemiological picture for the residents of the Serchio Valley, in particular for chronic kidney disease. In 2011, the Department of Statistics of the University of Florence conducted a descriptive analysis of the mortality and morbidity of residents in the 20 municipalities of the Serchio Valley (1996–2006) (42), which showed higher hospital-based mortality and higher prevalence of chronic-degenerative diseases than the regional average. Subsequently, this report was updated by the Regional Health Agency of Tuscany (ARS) with data for 1971–2015 for mortality and 1996–2017 for hospital admissions (43). The Regional Health Agency of Tuscany report found an excess of 10 deaths and 50 hospitalizations per year for urinary diseases, compared to the regional average for 2003–2017. The conclusions referred to the presence of heavy metals emissions in the area and other air and soil pollutants, for which there is evidence in the literature of an association with the diseases in excess in the area (44–46).

In addition, historical data from monitoring stations in the region provided further compelling evidence regarding cadmium exposure. The stations have recorded elevated levels of cadmium in the municipality of Barga dating back to the early 1980s, that likely originated from the deposition of airborne particulate matter released during the historical metallurgical activity that characterized the area. Moreover, a subsequent study conducted by geologists at the University of Pisa (21) confirmed the presence of heavy metals in the soil, particularly Cu, Zn and Cd.

Chronic exposure to environmental cadmium may have a toxic effect, causing both renal proximal tubular damage and eGFR decline (47, 48). The distribution of eGFR we found in the area could be explained by the long-lasting cadmium contamination of the soil and prolonged exposure of the population to heavy metals, including copper. This extended history of cadmium exposure suggests that the area’s environmental issues and health concerns are deeply rooted and have persisted for several decades.

Our study has several strengths. Namely, it allowed for the estimation of the prevalence of CKD stages in the area. In addition, it included subjects older than 18 years and with no upper age limit, allowing the inclusion of a wider population.

Another main strength of the study is the use of random sampling stratified by age and sex. Furthermore, DEGREE CKD definitions were used to facilitate international comparisons of CKD prevalence, help describe risk factors and identify the causes leading to CKD. From the public health perspective, we documented the existence of a hazard for those who worked in the local copper foundry and a risk for those who live close (<2 km) to the plant. Our results had public health impact on the procedures concerning the relocation of the air quality monitoring stations of the Environmental Protection Agency of Tuscany. An initiative is underway to characterize the contamination of the external environment by heavy metals.

However, our study has some potential limitations. Firstly, it could not show temporal associations between CKD and associated risk factors due to its cross-sectional design. In particular, for the temporal sequence, the duration of residence in the area or the years spent working in the copper foundry and the duration of the chronic illnesses (arterial hypertension or diabetes) should be further investigated. Moreover, only one eGFR measurement was carried out, so were not able to demonstrate chronicity and could not differentiate acute kidney injury (AKI) from CKD (49). Furthermore, the cross-sectional design inherently limited our ability to assess the role of potential confounders, such as dietary habits.

In addition, our study was conducted during the coronavirus-19 pandemic, which may have impacted the participation by the local population. Due to the low response rate, our sample size was smaller than that determined by a-priori power calculations. We noticed some differences in the response rate among the various age classes and by sex. However, we used post-stratification weights to reduce the sampling error and the potential non-response bias. Moreover, the overall sample size allowed us to achieve a good degree of accuracy for the estimation of the prevalence and it represents a good share of the reference population (see Supplementary Appendix A.1, Table A.1.1). We did not find evidence of a potential non response bias by location (see Supplementary Appendix A.4, Figure A.4.1).

CKD is considered to be a major public health problem worldwide (50, 51). Environmental pollutants, including heavy metals, can potentially increase the risk of CKD or accelerate its progression (52–54).

The present study aimed to identify the prevalence of CKD in the Serchio Valley, an area where the industrialization and the possible environmental impacts are a source of concern for the population’s health. Our results align with prior evidence in the literature, confirming an increased CKD risk in contexts characterized by exposure to environmental contaminants.

More specifically, our study confirmed an increased CKD risk for the population living in the Serchio Valley. In this study, diabetes, hypertension, work or residence near the copper foundry were found to be the factors most associated with decreased kidney function, although the precision of these risk estimates is limited due to the small sample size.

## Data Availability

The datasets presented in this article are not readily available because due to the sensitive nature of the data collected in this project, study participants were assured raw data would remain confidential and would not be shared. Requests to access the datasets should be directed to annibale.biggeri@unipd.it.
