# Supplementary material for: “Aria di Ricerca in Valle del Serchio”: a cross-sectional citizen science study to evaluate CKD prevalence and associations with environmental risk factors in the Serchio Valley (Lucca, Tuscany, Italy)
Source: Front Public Health. 2025 May 21;13:1536070. doi: 10.3389/fpubh.2025.1536070 (PMC12133754; doi:10.3389/fpubh.2025.1536070)
Supplement: Supplementary file 1 [file Data_Sheet_1.docx]

**"Aria di Ricerca in Valle del Serchio": a cross-sectional Citizen Science study to evaluate CKD prevalence and associations with environmental risk factors in the Serchio Valley (Lucca, Tuscany, Italy)**

**Appendix A**

- **A.1** Age and sex stratification of the reference population
- **A.2** Stratification of the planned sample by age and sex
- **A.3** Stratification of participants according to age and sex
- **A.4** Sampling density maps

**A.1 Age and sex stratification of the reference population**

**Table A.1.1.** Age and sex stratification of the reference population (residents in the municipality of Barga, according to the 2020 Census).

| Age classes (years) | Males | Females | Total |
| --- | --- | --- | --- |
| 18-39 | 995 | 974 | 1.969 |
| 40-49 | 668 | 705 | 1.373 |
| 50-59 | 781 | 768 | 1.549 |
| 60-69 | 626 | 635 | 1.261 |
| 70+ | 975 | 1.189 | 2.164 |
| Total | 4.045 | 4.271 | 8.316 |

**A.2 Stratification of the planned sample by age and sex**

**Table A.2.1** Age and sex stratification of the planned sample

| Age classes (years) | Males | Females | Total |
| --- | --- | --- | --- |
| 18-39 | 156 | 156 | 312 |
| 40-49 | 106 | 106 | 212 |
| 50-59 | 106 | 106 | 212 |
| 60-69 | 106 | 106 | 212 |
| 70+ | 56 | 56 | 112 |
| Total | 530 | 530 | 1060 |

**A.3 Stratification of participants according to age and sex**

**Table A.3.1.** Age and sex stratification of participants in the study

| Age classes (years) | Males | | Females | | Total | |
| --- | --- | --- | --- | --- | --- | --- |
|  | N | Response rate | N | Response rate | N | Response rate |
| 18-39 | 37 | 24% | 63 | 40% | 100 | 32% |
| 40-49 | 27 | 25% | 45 | 42% | 72 | 34% |
| 50-59 | 62 | 58% | 61 | 58% | 123 | 58% |
| 60=69 | 30 | 28% | 40 | 38% | 70 | 33% |
| 70+ | 22 | 39% | 13 | 23% | 35 | 31% |
| Total | 178 | 34% | 222 | 42% | 400 | 38% |

**A.4. Sampling Density Maps**

**(A) (B) (C)**


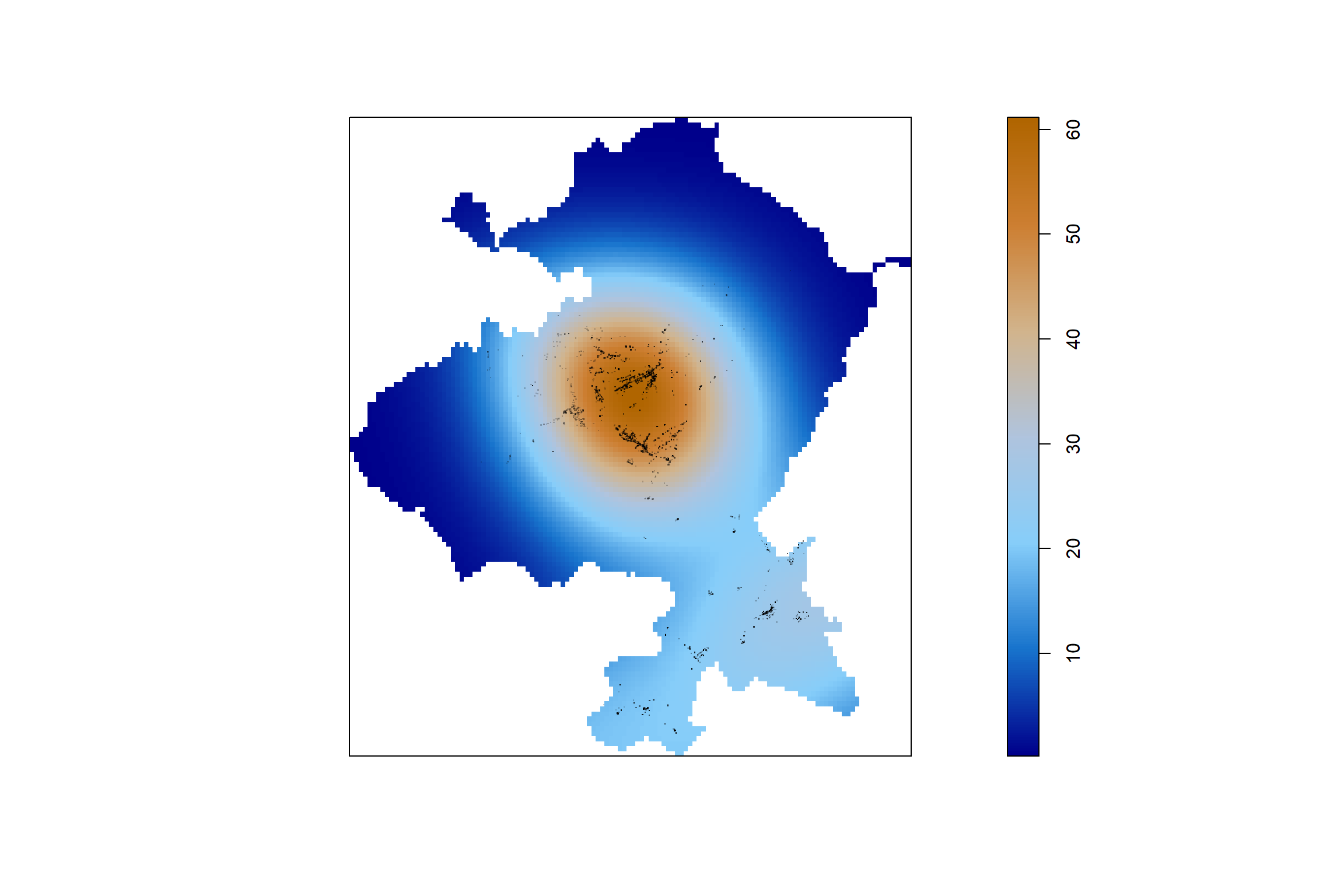

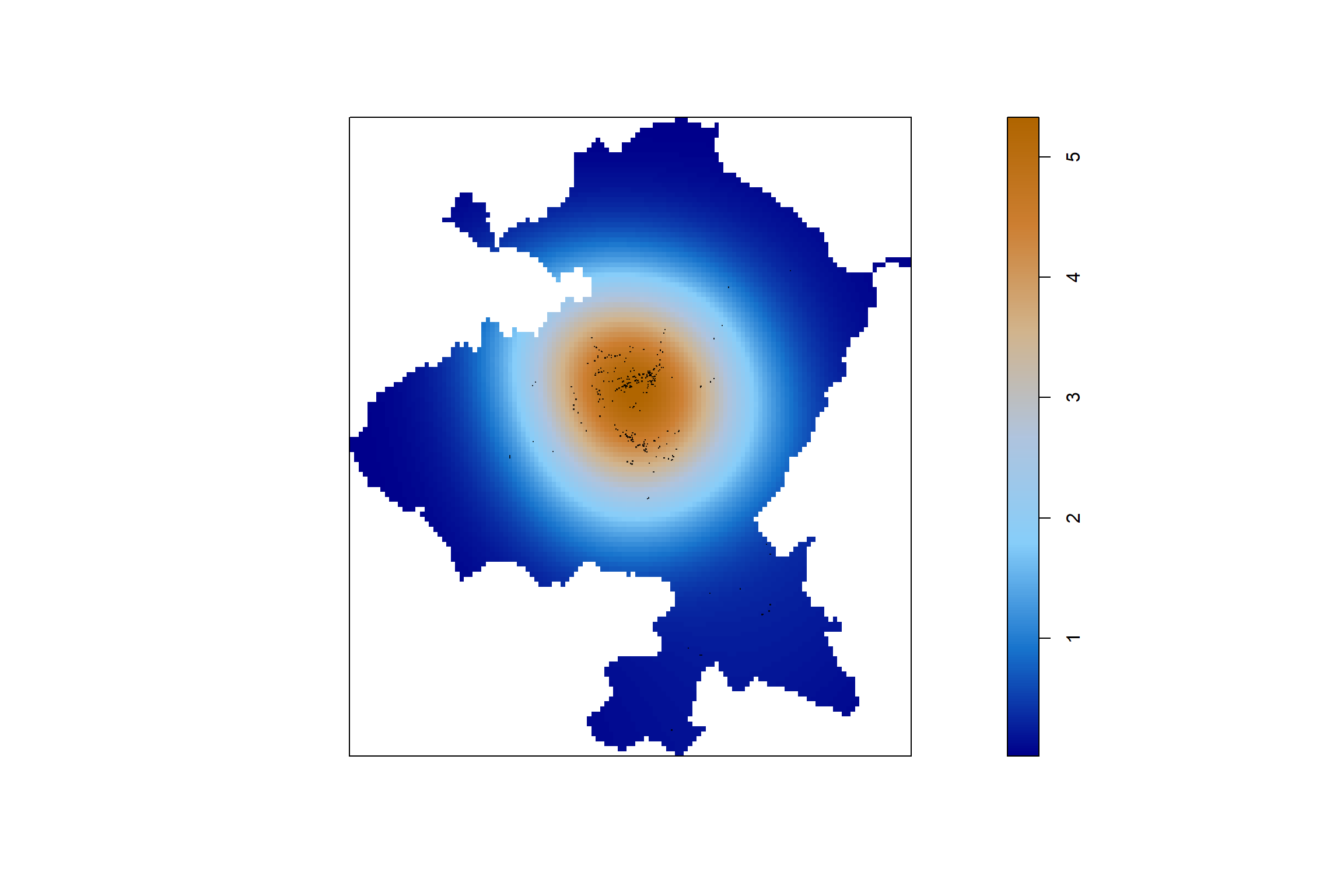

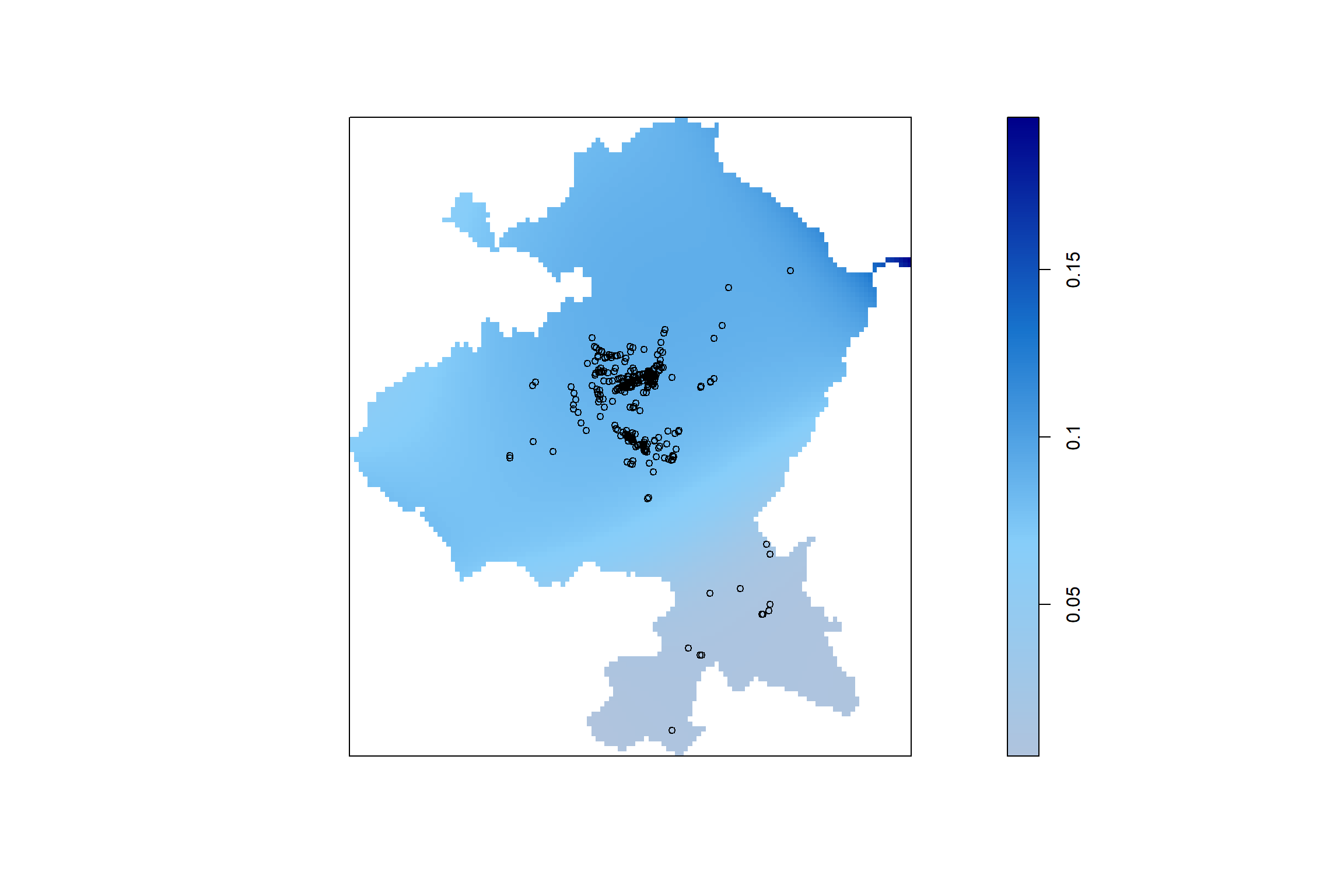


**Figure A.4.1.** Sampling density maps: **(A)** planned sample, (**B**) respondents and (**C**) ratio respondent/invited. Garfagnana, Tuscany Region (central Italy).
